# Can α-Mangostin and Photodynamic Therapy Support Ciprofloxacin in the Inactivation of Uropathogenic *Escherichia coli* and *Staphylococcus aureus* Strains?

**DOI:** 10.3390/ijms26010076

**Published:** 2024-12-25

**Authors:** Dorota Wojnicz, Kamila Korzekwa, Mateusz Guźniczak, Maciej Wernecki, Agnieszka Ulatowska-Jarża, Igor Buzalewicz, Dorota Tichaczek-Goska

**Affiliations:** 1 Department of Biology and Medical Parasitology, Faculty of Medicine, Wrocław Medical University, Mikulicza-Radeckiego 9, 50-345 Wroclaw, Poland; dorota.wojnicz@umw.edu.pl; 2 Department of Microbiology, Faculty of Biological Sciences, University of Wrocław, Przybyszewskiego 63, 51-148 Wroclaw, Poland; kamila.korzekwa@uwr.edu.pl (K.K.); maciej.wernecki2@uwr.edu.pl (M.W.); 3 BioQuanty Group, Department of Biomedical Engineering, Faculty of Fundamental Problems of Technology, Wroclaw University of Science and Technology, 27 Wybrzeże S. Wyspiańskiego St., 50-370 Wroclaw, Poland; mateusz.guzniczak@pwr.edu.pl (M.G.); agnieszka.ulatowska-jarza@pwr.edu.pl (A.U.-J.); igor.buzalewicz@pwr.edu.pl (I.B.)

**Keywords:** ciprofloxacin, α-mangostin, multidrug-resistant bacteria, PDT, UTI

## Abstract

Multidrug-resistant bacteria represent a significant challenge in the treatment of bacterial infections, often leading to therapeutic failures. This issue underlines the need to develop strategies that improve the efficacy of conventional antibiotic therapies. In this study, we aimed to assess whether a plant-derived compound, α-mangostin, and photodynamic therapy (PDT) could enhance the antibacterial activity of ciprofloxacin against uropathogenic strains of *Escherichia coli* and *Staphylococcus aureus*. Using nanopore sequencing technology, we confirmed that the clinical strains tested were classified as multidrug-resistant. Digital holotomography (DHT) was used to examine α-mangostin-induced changes in the bacterial cells’ penetration by a photosensitizer. A scanning confocal fluorescence microscope was used to visualize photosensitizer penetration into bacterial cells and validate DHT results. A synergistic effect between α-mangostin and ciprofloxacin was observed exclusively in *S. aureus* strains, while no enhancement of ciprofloxacin’s antibacterial activity was detected in *E. coli* strains when combined with α-mangostin. Notably, photodynamic therapy significantly potentiated the antibacterial effects of ciprofloxacin and its combination with α-mangostin compared to untreated controls. In addition, morphological changes were observed in bacterial cells exposed to these antimicrobials. In conclusion, our findings suggest that α-mangostin and PDT may serve as valuable adjuncts to ciprofloxacin, improving the eradication of uropathogens.

## 1. Introduction

The constant increase in drug resistance in microorganisms and the depletion of effective therapeutic resources have led to the development of alternative methods for treating infections. Ciprofloxacin is a broad-spectrum antibacterial drug commonly used in the treatment of UTIs. In recent years, strains resistant to fluoroquinolones, including ciprofloxacin, have been increasingly isolated. The acquisition of resistance by bacteria is a major cause of treatment failure and promotes the recurrence of UTIs [1]. Using natural compounds of plant origin or photodynamic therapy (PDT) for this purpose seems promising.

Mangostin, a natural polyphenolic compound belonging to the xanthone class, is predominantly found in the pericarp of mangosteen fruit (*Garcinia mangostana*) [2]. Its chemical structure features a xanthone core, which is responsible for its diverse biological activities. Mangostin exhibits a range of pharmacological properties, including antioxidant, anti-inflammatory, and anticancer effects [3,4]. However, its potent antibacterial and antibiofilm activities remain underexplored.

Existing studies indicate that mangostin has antibacterial properties against both Gram-positive and Gram-negative bacteria [5,6,7]. Gram-positive bacteria, such as *Staphylococcus aureus* and *Streptococcus pyogenes*, as well as Gram-negative bacteria, including *Escherichia coli* and *Pseudomonas aeruginosa*, are susceptible to its antimicrobial effects. However, most reported findings are based on antibiotic-sensitive bacterial strains, leaving its efficacy against drug-resistant strains largely unexplored. Our research is innovative because no studies have determined the interaction of ciprofloxacin, a plant-derived compound such as mangostin, and PDT on uropathogenic bacteria.

Mangostin has demonstrated efficacy in both inhibiting biofilm formation and disrupting preformed biofilms [8]. Biofilms are bacterial communities embedded in a protective extracellular matrix, which confer high resistance to antibiotics and host immune defenses. These structures are associated with chronic infections, including urinary tract infections (UTIs), contributing significantly to the persistence and recurrence of bacterial diseases. Because of the hydrophobic characteristics of α-mangostin, its bioavailability is quite limited [9]. However, research shows that α-mangostin has good antimicrobial activity against various pathogens such as *S. aureus* and *Enterococcus* sp. Accordingly, α-mangostin has been used as an adjuvant for antibiotics in the treatment of some infectious diseases [7,10,11].

Mangostin exerts its antibacterial effects through multiple mechanisms. It disrupts the bacterial cell membrane, causing leakage of intracellular contents and leading to cell death [5]. Additionally, it interferes with bacterial DNA replication and inhibits key enzymes involved in metabolic processes, further enhancing its bactericidal properties [12]. Mangostin also acts against biofilms by disrupting early-stage biofilm development, preventing bacterial adhesion to surfaces, and inhibiting the production of extracellular matrix components crucial for biofilm stability. Furthermore, it penetrates and disrupts mature biofilms, increasing bacterial susceptibility to conventional antibiotics and enhancing their clearance by the host immune system [13,14]. The increasing antibiotic resistance of uropathogenic bacteria poses a significant challenge in healthcare, necessitating the development of safe and effective alternatives to traditional treatments. PDT is a promising approach for targeting a wide range of microorganisms. PDT employs photosensitizers that, upon light activation, generate reactive oxygen species (ROS) [15]. These ROS inhibit bacterial cell division, inactivate pathogens, and disrupt various metabolic pathways [16].

The mechanism of PDT relies on the ROS generated after the photosensitizer is exposed to activating light. These ROS inflict extensive damage to the cytoplasmic membrane of microbial cells, causing leakage of intracellular components, disruption of transmembrane transport, inhibition of enzymatic activities, and DNA damage [16].

The emergence and continued rise of antibiotic-resistant bacteria have necessitated the development of novel treatment strategies. Mangostin has demonstrated the ability to synergize with conventional antibiotics, enhancing their antibacterial activity and potentially reducing the likelihood of resistance development [17]. Additionally, combining mangostin with other natural compounds has shown promising results in combating bacterial infections [18]. It was found that mangostin enhances the efficacy of aminolevulinic acid-PDT against cancers, e.g., gastric cancer [19].

The objective of our study was to evaluate the effects of ciprofloxacin (CIP), α-mangostin (M), and their combination (CIP + M) on the growth of both antibiotic-sensitive and multidrug-resistant strains of *S. aureus* and *E. coli*, and to assess the occurrence of posttreatment effects. Since both α-mangostin and ciprofloxacin inhibit bacterial DNA replication as part of their mechanisms of action, we also investigated whether PDT, which disrupts DNA functioning through ROS, could enhance the activity of these compounds. Notably, no existing studies have examined the synergistic potential of α-mangostin in combination with PDT against bacterial pathogens, underscoring the novelty of this investigation.

## 2. Results

### 2.1. Antimicrobial Susceptibility Testing

The resistance profiles revealed that both clinical strains have MDR profiles [20]. MRSA 101 was resistant to ciprofloxacin, erythromycin, clindamycin, gentamicin, and cefoxitin but susceptible to trimethoprim/sulfamethoxazole and vancomycin. *E. coli* 208 was resistant to amoxicillin-clavulanic acid, cephalexin, cefuroxime, cefotaxime, ceftazidime, ciprofloxacin, and trimethoprim/sulfamethoxazole but susceptible to amikacin, imipenem, meropenem, and piperacillin-tazobactam.

### 2.2. Identification of Antibiotic Resistance Genes

The antibiotic resistance genes found in the tested strains, listed with the closest matches in GenBank and their identity values, are shown in Table 1. Antibiotic resistance genes were found in *S. aureus* ATCC 29213, MRSA 101, and *E. coli* 208. The reference *E. coli* ATCC 25922 strain does not have antibiotic resistance genes.

### 2.3. MIC Evaluation

The MIC values of ciprofloxacin and α-mangostin are presented in Table 2. According to the EUCAST criteria [21], the MIC values of ciprofloxacin (128 μg/mL and 64 μg/mL) against two clinical strains, MRSA 101 and *E. coli* 208, indicated that the tested isolates were resistant to this antibiotic. The MIC values of ciprofloxacin (0.5 μg/mL and 0.015 μg/mL) against the reference *S. aureus* ATCC 29213 and *E. coli* ATCC 25922 strains allowed these isolates to be classified as ciprofloxacin-susceptible. The MICs of α-mangostin were significantly greater than those of ciprofloxacin and were 512 μg/mL or 64 μg/mL (Table 2).

### 2.4. Effect of the Ciprofloxacin and α-Mangostin Combination on Bacterial Survival

**Figure 1:**
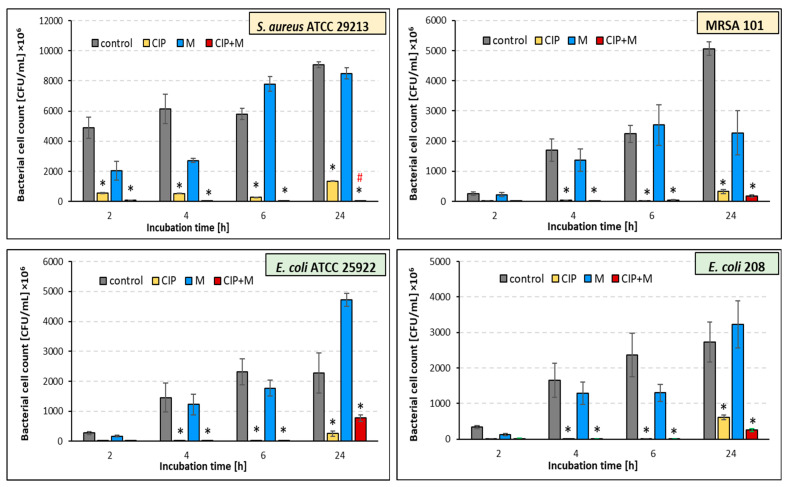
Anti-growth effects of ciprofloxacin (CIP), α-mangostin (M), and their combination (CIP + M) against *S. aureus* ATCC 29213, *E. coli* ATCC 25922, MRSA 101, and *E. coli* 208. The values represent the means from three independent experiments. Statistically significant differences between samples containing CIP, M, CIP + M, and untreated samples (controls) are marked with an asterisk (* *p* ≤ 0.05). The “red hash” (#) indicates a statistically significant reduction in the survival of doubly treated bacteria (CIP + M) compared to single treatment with CIP (# *p* ≤ 0.05).

To determine the antibacterial effect of ciprofloxacin and α-mangostin, we examined whether incubation of bacteria with these antimicrobials would result in their reduced survival compared to untreated bacteria (controls) (Figure 1). The results of our research indicate good antigrowth activity of ciprofloxacin against all tested strains during the whole incubation period (*p* ≤ 0.05). There are few reports on the antibacterial activity of α-mangostin, so we decided to check the effect of this plant metabolite on planktonic cultures. Our studies indicate a weak antibacterial activity of α-mangostin. Compared with the control samples, the number of viable cells of both Gram-positive strains (*S. aureus* ATCC 29213 and MRSA 101) decreased under the α-mangostin single treatment, except for the 6 h cultures (Figure 1).

In contrast, in the case of Gram-negative strains (*E. coli* ATCC 25922 and *E. coli* 208), the antigrowth effect of α-mangostin lasted only for 6 h (Figure 1), meaning only in the early stages of growth of these bacteria. Notably, in the 24 h α-mangostin-treated *E. coli* cultures, bacterial growth was, unfortunately, better than in the untreated samples. Due to the relatively weak effect of α-mangostin when administered alone, we checked whether it could support the antibacterial effect of ciprofloxacin, i.e., act synergistically with this chemotherapeutic agent. To determine the synergistic effect of ciprofloxacin and α-mangostin, bacteria were incubated in a medium containing both antimicrobials (double treatment). The results were compared to bacteria incubated with ciprofloxacin alone.

A synergistic effect of α-mangostin with ciprofloxacin was noted for both tested *S. aureus* strains, regardless of the incubation time (Figure 1). However, a statistically significant reduction in staphylococci growth was noticed only in the 24 h culture of the reference strain (Figure 1; “red hash”; *p* ≤ 0.05). Unfortunately, the synergistic effect of α-mangostin with ciprofloxacin was reached against neither the *E. coli* ATCC 25922 nor the *E. coli* 208 strains (Figure 1). An analysis of the results suggests that α-mangostin exhibited a slight antagonistic effect on the activity of ciprofloxacin against the *E. coli* ATCC 25922 strain.

### 2.5. Estimation of Post-Treatment Duration Time Effects of Ciprofloxacin, α-Mangostin, and Their Combination

**Figure 2:**
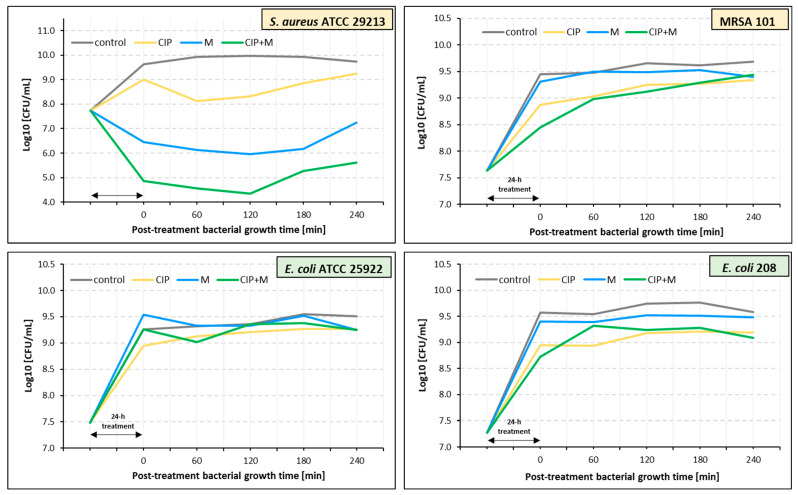
Post-treatment duration time effects (PTEs) of ciprofloxacin (CIP), α-mangostin (M), and their combination (CIP + M) against *S. aureus* ATCC 29213, *E. coli* ATCC 25922, MRSA 101, and *E. coli* 208. The values represent the means from three independent experiments.

A post-treatment effect (PTE) of ciprofloxacin, α-mangostin, and their combination against *S. aureus* ATCC 29213 and *E. coli* ATCC 25922 was observed (Table 3, Figure 2). PTE times were longer for *S. aureus* ATCC 29213 than for *E. coli* ATCC 25922 regardless of the antibacterial agent. Ciprofloxacin and α-mangostin had the longest PTE duration, lasting 195 min for *S. aureus* ATCC 29213. In the case of *E. coli* ATCC 25922, the longest PTE (180 min) was noted for α-mangostin.

Ciprofloxacin, α-mangostin, and their combination did not exhibit PTE for MRSA 101 (Figure 2, Table 3). A similar result was obtained for *E. coli* 208, except for ciprofloxacin, which showed a short-term PTE lasting only 60 min.

### 2.6. α-Mangostin-Induced Changes in Bacterial Cells’ Penetration by a Photosensitizer

**Figure 3:**
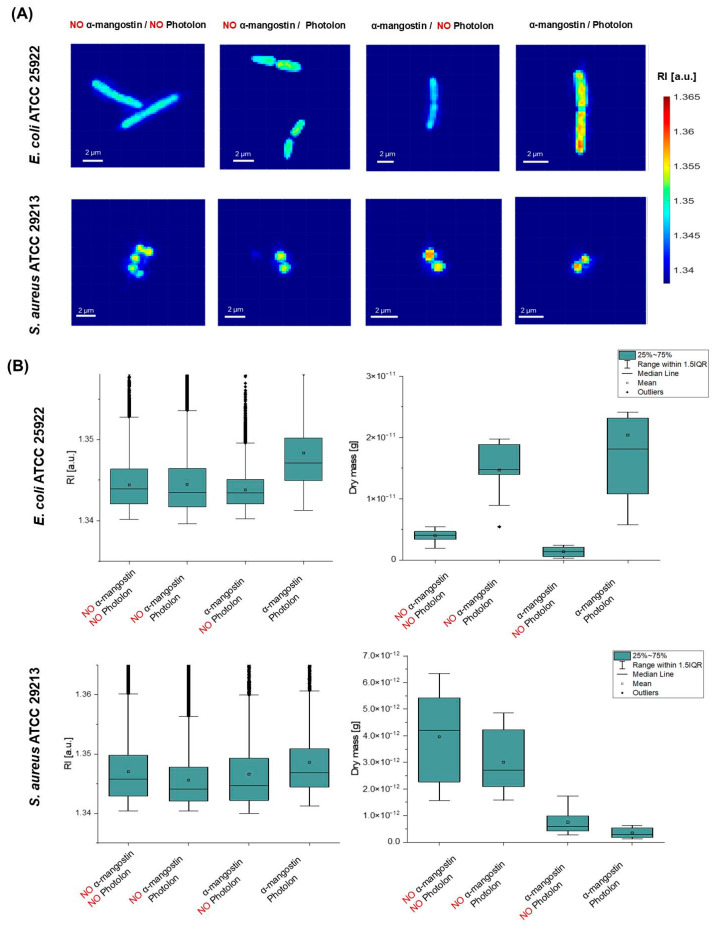
Representative DHT results: (**A**) 2D-RI maps and (**B**) box plots of the average RI and dry mass of *E. coli* ATCC 25922 and *S. aureus* ATCC 29213 bacterial cells that were untreated (NO α-mangostin/NO Photolon) or treated (by NO α-mangostin/Photolon, by α-mangostin/NO Photolon, and by both α-mangostin/Photolon).

Digital holotomography (DHT) was used to obtain the 3D distribution of the refractive index (RI) (so-called 3D RI tomogram) of bacterial cells. This quantity is directly related to the density of the cells, so any local changes in density caused by changes in the permeability of the cell membrane and the accumulation of the photosensitizer inside the cell will be revealed by the change in the average RI value of the cell. Representative RI-based results are shown in Figure 3.

In the case of *E. coli* ATCC 25922, the 2D RI maps of individual bacterial cells show that a significant increase in RI values is observed for cells treated with both α-mangostin and Photolon, while the range of changes in RI values is comparable in the case of untreated cells (no α-mangostin, no Photolon) or cells treated with α-mangostin or Photolon separately (Figure 3A). This may indicate that Photolon and α-mangostin used separately did not induce changes in cell density and did not accumulate inside the cells. Based on the analysis performed, it was possible to determine the range of variation of RI values within the region occupied by a single cell. In the case of *E. coli* ATCC 25922, it was 1.34018–1.3748 for untreated cells, 1.3396–1.3759 for cells treated with Photolon, 1.3402–1.3579 for cells treated with α-mangostin, and 1.3413–1.39228 for cells treated with both α-mangostin and Photolon. Analysis of the box plots (Figure 3B) representing the variation in RI values shows that a significant increase in RI values is observed for cells treated with α-mangostin and Photolon, indicating a local increase in density within these cells. The median RI values for untreated cells, cells treated with Photolon, cells treated with α-mangostin, and cells treated with the combination of Photolon and α-mangostin are 1.34392, 1.34346, 1.34344, and 1.34711, respectively. The one-way ANOVA confirms that, at the 0.05 significance level, there are statistically significant changes in RI values between *E. coli* ATCC 25922 cells treated with Photolon alone and with the combination of Photolon and α-mangostin. Furthermore, the box plots representing the variation of the dry mass value of individual cells (Figure 3B) indicate that the use of the Photolon photosensitizer leads to an increase in the density and volume of individual cells, which can be explained by the accumulation of the photosensitizer inside the cells. However, this effect is much more significant for the combination of α-mangostin and Photolon, which leads to a more significant increase in the dry mass value of the bacterial cells, which may indicate that α-mangostin promotes more effective penetration of the bacterial cells by Photolon. This suggests that α-mangostin may promote the accumulation of photosensitizers inside the cell.

In the case of S. *aureus* ATCC 29213, representative 2D-RI maps of individual bacterial cells show that no significant increase in RI values in comparison with the untreated cells is observed for cells treated by Photolon, but in the case of cells treated by α-mangostin or the combination of α-mangostin and Photolon, an increase in RI values is observed inside the region occupied by the bacterial cells (Figure 3A). This suggests that Photolon itself does not accumulate in cells. Moreover, the application of α-mangostin leads to a decrease in the size of S. aureus cells. The range of variation of RI values was 1.3404–1.3756 for untreated cells, 1.3404–1.3781 for cells treated with Photolon, 1.3399–1.3755 for cells treated with α-mangostin, and 1.3412–1.3748 for cells treated with both α-mangostin and Photolon. A more complex analysis can be performed based on the box plots (Figure 3B) representing the variation of RI values inside the bacterial cells in examined samples. It was shown that the median RI value of cells treated with Photolon (1.34326) was slightly lower than in untreated cells (1.3454), suggesting that the photosensitizer did not penetrate the *S. aureus* ATCC 29213 cells. A similar behavior is observed for α-mangostin, for which the median RI value is 1.3454. On the other hand, in the case of cells treated with the combination of α-mangostin and Photolon, as the median of the RI value is equal to 1.34685 and is higher than in the case of untreated cells and samples treated separately with Photolon and α-mangostin. The one-way ANOVA confirms that, at the 0.05 significance level, there are statistically significant changes in RI values between *S. aureus* cells treated with Photolon alone and cells treated with the combination of Photolon and α-mangostin. The increase in the median of the RI values indicates an increase in the density of the cells, which may be related to the accumulation of Photolon inside the cell. However, it should be noted that this effect is weaker than in the case of *E. coli* ATCC 25922 cells. Moreover, the box plots of the variation of the dry mass of the cells showed that the application of α-mangostin induced a significant reduction in the dry mass of bacterial cells in comparison with the untreated samples and those treated only with Pholoton. Compared to the median dry mass of untreated cells (4.20123 *×* 10^−12^ g) and cells treated with Photolon (2.70358 × 10^−12^ g), the median dry mass of cells treated with α-mangostin alone and with a combination of α-mangostin and Photolon is equal to 5.84707 × 10^−13^ g and 2.94009 × 10^−13^ g, respectively. These changes in dry mass can be explained by the α-mangostin-induced reduction in bacterial cell size, leading to a reduction in cell volume, which in turn leads to a reduction in bacterial cell dry mass. The obtained results suggest that α-mangostin may promote the accumulation of Photolon inside *S. aureus* ATCC 29213 cells; however, this effect is weaker than in the case of *E. coli* ATCC 25922.

**Figure 4:**
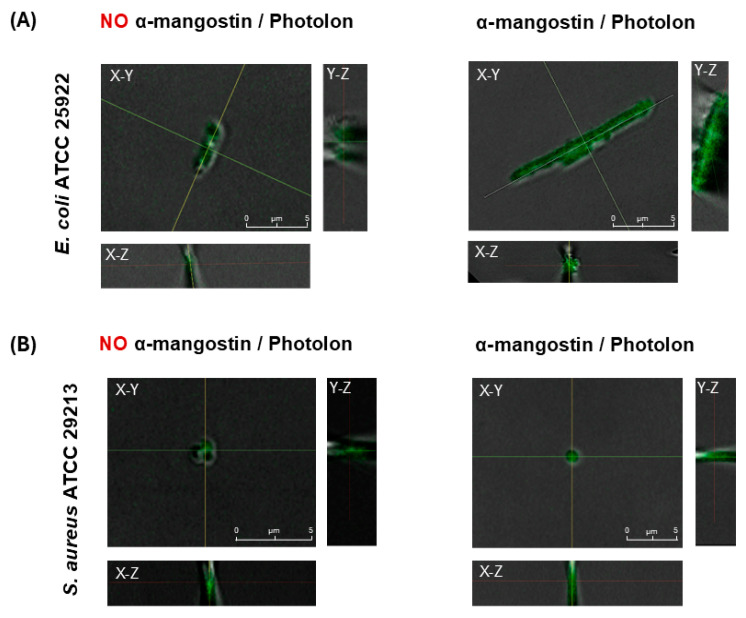
Representative confocal 2D microscopic images (combined DIC-fluorescence images) of *E. coli* cells (**A**) penetrated by Photolon (left column) and a combination of Photolon and α-mangostin (right column), and *S. aureus* cells (**B**) penetrated by Photolon (left column) and a combination of Photolon and α-mangostin (right column); (lines indicate the planes for which the axial (X-Z, Y-Z) cross-sections were extracted).

The bacterial samples were examined by confocal microscopy to confirm the lack of autofluorescence of the bacteria itself. The photophysical properties of α-mangostin do not show excitation sensitivity at wavelengths greater than 400 nm [22]; therefore, samples of bacteria incubated with Photolon alone and with Photolon and α-mangostin were imaged. After photoexcitation of the Photolon by laser light at a wavelength equal to 405 nm, which corresponds to the Soret band [23], confocal microscopic images were registered, showing the interaction of the bacterial cells with Photolon and α-mangostin (Figure 4).

The analysis of the differential interference contrast (DIC) and fluorescence images of cells, as well as axial cross-sections (X-Z, Y-Z), resulted in the observation that the fluorescence signal is spatially overlapping in all regions occupied by the bacterial cells—not only in the cells’ capsule, cell wall, or cytoplasm membrane but also inside of the cells of both Gram-positive and Gram-negative bacteria species. The results indicate the presence of Photolon inside the cells, which confirms the ability of the used photosensitizer to penetrate through the bacterial cell wall into the cell of both examined species (reference strains *E. coli* ATCC 25922 and *S. aureus* ATCC 29213). Anionic photosensitizers such as Photolon are not taken up into bacterial cells via simple diffusion. Uptake of anionic photosensitizers may be mediated through a combination of electrostatic charge interactions and protein transporters. Efflux pumps are bacterial transport proteins that are involved in the extrusion of substrates from the cellular interior to the external environment. Mangostin is a natural product that has been identified as an efflux pump inhibitor.

Recent studies revealed that α-mangostin was associated with changes in membrane permeability and inhibition of the *MepA* and *NorA* genes, which encode the efflux pumps of MRSA [24]. It was proven that it is possible to photodynamically inactivate Gram-negative bacteria without photosensitizer accumulation in the bacterial cells. This fact is especially interesting considering that the development of resistance may be prevented, leaving active components such as Photolon outside of the bacterium. However, the fluorescence intensity of the obtained images indicates that the penetration of both bacteria species’ cells is more effective in the case of Photolon assisted by α-mangostin. Moreover, the comparison of the fluorescence signal intensity (Figure 4, main panel) revealed that the concentration of Photolon with α-mangostin inside the cells of *S. aureus* is lower than in *E. coli* in the case of Photolon itself, which indicates a lower penetration of the cell by this composition or by the mutual interaction of both substances.

These observations were also confirmed by the analysis of the axial fluorescence intensity of single *E. coli* and *S. aureus* cells (Figure 4, side and lower panels), demonstrating a higher fluorescence intensity in the case of *E. coli*, which corresponds to the accumulation and concentration of Photolon inside the cells. These results are consistent across the changes in the RI of examined cells because, for *S. aureus*, the fluorescence intensity was significantly lower than for *E. coli*. Therefore, the high correlation of the changes in the RI value of the cells, along with the efficiency of the cells’ penetration by α-mangostin and Photolon, proved the capability of digital holotomography in the characterization of local density changes in single cells. The proposed combination would maximize the efficiency of α-mangostin in a PDT or drug delivery system. Thus, extensive studies on α-mangostin-based trials may benefit the approach of novel therapies for managing antimicrobial strategies.

### 2.7. Effect of PDT on Bacteria Pre-Treated with Ciprofloxacin, α-Mangostin, and Ciprofloxacin with α-Mangostin

**Figure 5:**
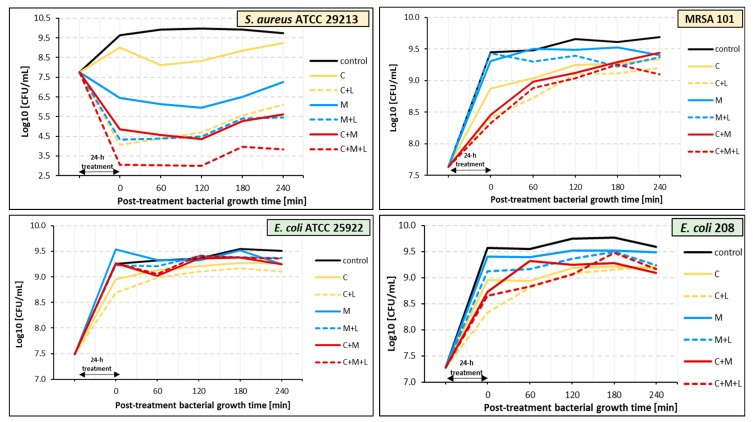
Effect of PDT on *S. aureus* ATCC 29213, *E. coli* ATCC 25922, MRSA 101, and *E. coli* 208 pre-treated with ciprofloxacin (C), α-mangostin (M), and ciprofloxacin with α-mangostin (C + M). The values represent the means from three independent experiments.

In the last step of our research, we examined whether PDT can enhance the antibacterial effect of ciprofloxacin (C + L), α-mangostin (M + L), and ciprofloxacin combined with α-mangostin (C + M + L). The impact of PDT on bacterial strains after 24 h of pre-treatment with the compounds and mixtures of compounds mentioned above is shown in Figure 5. The greatest antibacterial effect of PDT was observed against *S. aureus* ATCC 29213. PDT applied to the samples containing bacteria pre-treated with ciprofloxacin (C + L) resulted in a growth reduction of 3.2–4.9 compared to non-irradiated control samples. A statistically significant decrease in *S. aureus* ATCC 29213 survival persisted until 180 min after irradiation (*p* ≤ 0.05). Irradiation of the samples containing *S. aureus* ATCC 29213 bacteria pre-treated with α-mangostin (M + L) and the combination of ciprofloxacin and α-mangostin (C + M + L) resulted in a growth reduction of 1.1–2.1 log10 and 1.3–1.8 log10, respectively (Figure 5) (*p* > 0.05).

In the case of the other irradiated strains, the effect of PDT was much weaker (Figure 5). After irradiation, the number of viable bacterial cells in the tested suspensions was reduced by 0.1–0.6 log_10_, compared to non-irradiated samples. The most effective light-mediated killing (0.6 log_10_ reduction for *E. coli* 208 and 0.5 log_10_ reduction for *S. aureus* MRSA) was revealed in samples pre-treated with ciprofloxacin (C + L). This effect was observed immediately after irradiation (t0) and then weakened (Figure 5). Although the reduction in survival of *E. coli* 208 and MRSA 101 was weak compared to their non-irradiated counterparts, data analysis revealed that PDT enhanced the antibacterial activity of ciprofloxacin as well as α-mangostin and their combination when compared to the control (*p* ≤ 0.05), a difference that could not be observed in non-irradiated samples.

### 2.8. Effect of Ciprofloxacin, α-Mangostin, and PDT on the Morphology of Bacterial Cells

**Figure 6:**
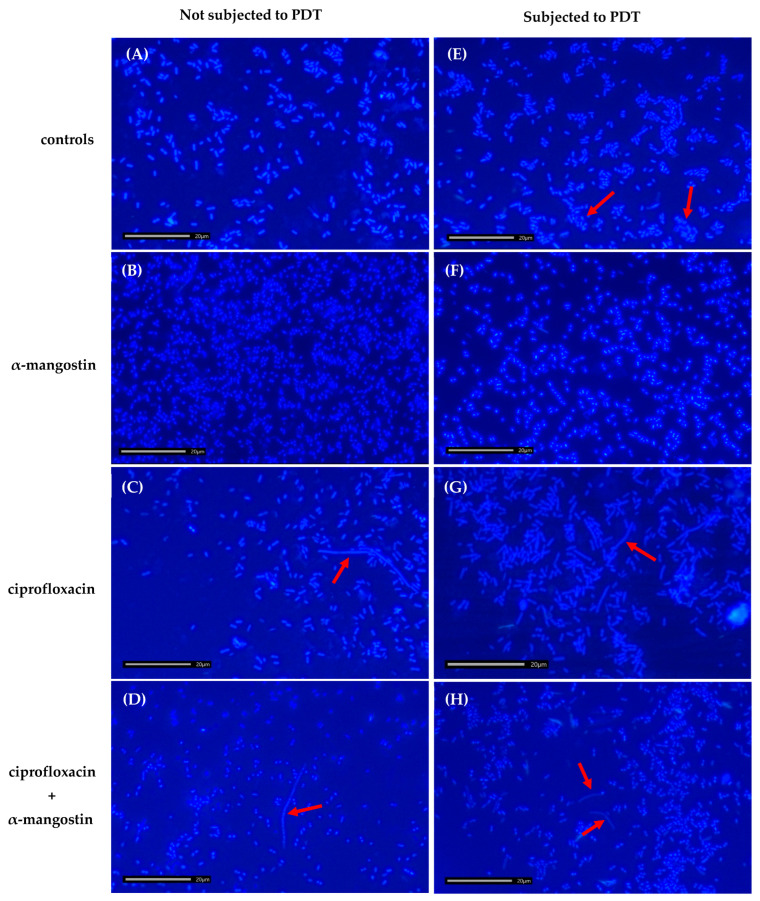
Fluorescence microscopy images demonstrating alterations in the morphology of bacterial cells (DAPI staining; magnification ×1000). In samples (**B**,**F**), bacterial cells were smaller than in the controls (**A**,**E**); long filaments were visible (**C**,**D**,**G**); so-called “ghost” cells partially lacking a cell wall were observed (**H**); and bacteria subjected to PDT formed clusters (**E**–**H**). Morphological changes are indicated with red arrows.

In the control sample, the bacteria measured 2–3 µm in size and were observed as individual cells, without forming clusters (Figure 6A). In bacterial cultures treated with α-mangostin, *E. coli* cells were slightly smaller than those in the control (Figure 6B,F). In samples treated with ciprofloxacin, long filaments exceeding 15 µm in length were observed (Figure 6C,G). In all cultures subjected to PDT, bacterial cells were observed to form clusters (Figure 6E–H). Additionally, in PDT-subjected samples containing α-mangostin and ciprofloxacin, so-called “ghost” cells partially lacking a cell wall were observed (Figure 6H).

The formation of filaments in samples containing ciprofloxacin is attributed to its mechanism of action, which involves inhibiting bacterial cell division by blocking DNA synthesis. The bacteriostatic effect arises from the inhibition of DNA and RNA synthesis due to the formation of the gyrase–DNA–CIP complex. This complex may interfere with the expression of genes responsible for septum formation. It is hypothesized that abnormal elongation occurs when the antibiotic concentration is sufficient to inhibit the synthesis of enzymes required for septation but insufficient to impede those involved in elongation. Mangostin disrupts bacterial envelopes, leading to the leakage of intracellular contents. This disruption inhibits bacterial growth and division, ultimately resulting in cell death. Consequently, bacterial cells cultured in the presence of α-mangostin may exhibit reduced cell length compared to untreated bacteria. PDT utilizes photosensitizing compounds to generate ROS within bacterial cells. These highly reactive molecules cause damage to the bacterial envelope, proteins, and nucleic acids, ultimately causing cell death. Furthermore, the damage to the bacterial envelope and subsequent leakage of intracellular contents can promote cell aggregation, potentially explaining the cluster formations observed under fluorescence microscopy.

## 3. Discussion

Modern medicine faces a serious challenge—the growing resistance of bacteria to antibiotics. Multidrug-resistant bacterial strains, such as *S. aureus* and *E. coli*, represent significant global health threats [25]. As traditional antibiotics become increasingly ineffective, researchers are exploring alternative therapeutic strategies. One promising approach involves combining conventional antibiotics with natural compounds that enhance their efficacy. Among these, α-mangostin—a bioactive plant-derived compound—has garnered attention for its diverse biological activities, including antibacterial, anti-inflammatory, and antioxidant effects [26,27,28,29].

Our results confirmed the antigrowth activity of α-mangostin against both antibiotic-sensitive strains (*S. aureus* ATCC 29213 and *E. coli* ATCC 25922) and multidrug-resistant strains (MRSA 101 and *E. coli* 208). Laboratory studies have demonstrated that α-mangostin exhibits strong activity against various pathogens, including antibiotic-resistant strains [10,11,30,31]. Notably, α-mangostin has shown potent antibacterial properties against resistant bacteria such as *E. coli*, MRSA, and vancomycin-resistant *Enterococci* (VRE) [7,10,11,32,33,34]. Its antibacterial efficacy has also been observed against other pathogens, including *Staphylococcus epidermidis*, *S. aureus*, *Streptococcus pyogenes*, *Propionibacterium acnes*, *Pseudomonas aeruginosa*, and *Klebsiella pneumoniae* [17,30,31,35]. Additionally, α-mangostin has been reported to be more effective against Gram-positive than Gram-negative bacteria [10]. This differential activity is attributed to the outer membrane of Gram-negative bacteria, which acts as a barrier to hydrophobic mangostin molecules.

The structural differences in the outer cell envelopes of Gram-positive and Gram-negative bacteria play a crucial role in the penetration of photosensitizers. In Gram-negative bacteria, the presence of an outer membrane poses a significant barrier to photosensitizer entry. This membrane is enriched with lipopolysaccharides, which are hydrophobic and form a protective layer that restricts the access of hydrophilic compounds. Additionally, the outer membrane contains porin proteins that selectively permit the passage of small, hydrophilic molecules, further impeding the penetration of larger or hydrophobic compounds. Another challenge is the highly negative surface charge of Gram-negative bacteria, which repels many photosensitizers. To enhance photosensitizer effectiveness, including penetration of the outer membrane, conjugation with cationic compounds or nanoparticles has been employed. For instance, Wang et al. [36] developed a conjugate of chlorin-e6 with the cation 1-vinyl-3-dodecylimidazole, which improved the efficacy of antimicrobial photodynamic therapy against *E. coli* and *S. aureus*. Similarly, Le Guern et al. [37] designed CNCs_c6-PMB_, a photobactericidal organic material comprising cellulose nanocrystals functionalized with the photosensitizer chlorin-e6 and the antimicrobial polypeptide polymyxin B (PMB). These modified nanocrystals demonstrated effective photobacterial activity against both Gram-negative bacteria (*E. coli* and *P. aeruginosa*) and Gram-positive bacteria (*S. aureus* and *S. epidermidis*).

Two multidrug-resistant strains, MRSA 101 and *E. coli* 208, both of which are resistant to ciprofloxacin, were utilized in our studies. Ciprofloxacin, a widely used antibiotic from the fluoroquinolone group, is commonly prescribed to treat bacterial infections, including those of the urinary tract, respiratory system, digestive system, and skin. Its mechanism of action involves inhibiting bacterial enzymes—topoisomerase IV and DNA gyrase—both essential for DNA replication [38]. By blocking these enzymes, ciprofloxacin prevents bacterial reproduction. However, over the years, bacteria have developed resistance mechanisms against ciprofloxacin, necessitating ongoing research to enhance its efficacy. One promising method involves combining ciprofloxacin with plant-based substances to achieve synergistic effects. Synergy occurs when the combination of two substances produces an effect greater than the sum of their individual effects. Since α-mangostin inhibited the growth of the tested multidrug-resistant bacteria, we investigated whether it exhibited synergistic activity with ciprofloxacin against these strains. A synergistic effect between α-mangostin and ciprofloxacin was observed for MRSA 101, irrespective of incubation time. For the *E. coli* 208 strain, this effect was evident only after 24 h of incubation.

In vitro studies have demonstrated that α-mangostin can enhance bacterial sensitivity to ciprofloxacin by destabilizing the bacterial cell membrane [10,39]. This membrane disruption likely facilitates the penetration of the antibiotic into bacterial cells, increasing its intracellular concentration at the target site and thereby boosting its antimicrobial efficacy [39]. Additionally, α-mangostin may interfere with bacterial defense mechanisms, such as inhibiting efflux pumps, which are a major resistance mechanism against ciprofloxacin [12].

The available literature indicates that α-mangostin exhibits synergistic effects with various antibiotics, enhancing their efficacy against resistant strains [11,17,40,41]. Ahmad et al. [17] reported a synergistic effect between α-mangostin and antibiotics such as erythromycin, tetracycline, and clindamycin against multidrug-resistant bacteria associated with acne, including *P. acnes*, *S. aureus*, *S. epidermidis*, and *S. pyogenes*. Their study found that combining α-mangostin with these antibiotics effectively inhibited bacterial growth after 10–12 h of incubation. Similarly, Phitaktim et al. [40] observed a synergistic effect between α-mangostin and oxacillin against oxacillin-resistant *S. saprophyticus*. This combination significantly reduced bacterial cell counts within 4 to 24 h of incubation. These findings align with those of Sakagami et al. [11], who demonstrated that α-mangostin, when combined with gentamicin or vancomycin hydrochloride, exhibited synergistic activity against VRE and MRSA, respectively. The synergistic effects of α-mangostin and antibiotics in broadening the “antimicrobial spectrum” were evaluated by Suksamrarn et al. [41]. They reported that α-mangostin exhibited a strong inhibitory effect against *Mycobacterium tuberculosis* when tested alongside standard drugs such as rifampicin, isoniazid, and kanamycin. In contrast, Ibrahim et al. [42] found that combining α-mangostin with ampicillin, penicillin G, tetracycline, or streptomycin did not produce antibacterial activity against *E. coli* or *S. aureus.* Phitaktim et al. [40] proposed that the antibacterial action of α-mangostin follows a three-step process. First, it disrupts the cytoplasmic membrane, increasing its permeability. Next, bacterial enzymes involved in cell wall synthesis become inhibited. Finally, the peptidoglycan structure has been damaged, compromising the bacterial cell wall. Supporting these findings, Koh et al. [10] demonstrated that the isoprenyl groups of α-mangostin play a critical role in penetrating the lipid bilayer of the MRSA cell membrane, leading to membrane disintegration and increased permeability. Furthermore, α-mangostin has been shown to depolarize the bacterial cell membrane, inducing approximately 37% leakage due to vesicle lysis within 5–10 min [10].

One of the major challenges associated with long-term antibiotic use is the increased risk of bacterial resistance. Combining ciprofloxacin with mangostin can reduce this risk. Due to their synergistic effect, lower ciprofloxacin doses can be used while maintaining high therapeutic efficacy. Reducing antibiotic dosage lowers the selection pressure on bacteria, decreasing the probability of resistant strains emerging.

Most studies indicate synergistic effects between plant metabolites and antibiotics, which has also been confirmed by the results of our research. However, in a 24 h culture of *E. coli* ATCC 25922, we observed an antagonistic interaction between α-mangostin and ciprofloxacin (Figure 1). During the initial hours (2, 4, and 6 h), both compounds demonstrated synergistic activity. The plant metabolite may have increased the permeability of the bacterial cell membrane, facilitating the penetration of the antibiotic and enhancing its efficacy. Over prolonged exposure (24 h), bacteria may activate adaptive mechanisms, such as efflux pumps that remove both substances, thereby reducing their intracellular concentration and effectiveness. Certain plant-derived secondary metabolites, such as salicylic acid, influence bacterial regulatory systems such as the marRA and soxRS systems [43]. These systems regulate the expression of efflux pumps, such as the AcrAB–TolC system, which contributes to multidrug resistance. For instance, salicylic acid can activate marA, indirectly enhancing the efflux pump’s ability to expel antibiotics, such as colistin and other cationic antimicrobial peptides, thereby reducing their intracellular concentration [43]. The antagonistic interaction between mangostin and ciprofloxacin may result from the formation of shared complexes that hinder ciprofloxacin from reaching its target site, the enzyme DNA gyrase. A similar relationship has been observed between the plant metabolite caffeine and erythromycin. The complexes formed likely prevented both agents from reaching their respective target sites [44].

Research has also shown that mangostin derivatives can be inhibitors of bacterial efflux pumps [24]. Efflux pumps are mechanisms that bacteria use to expel antibiotics and other harmful compounds, playing a significant role in antimicrobial resistance. By inhibiting these pumps, mangostin derivatives can restore the efficacy of antibiotics against multidrug-resistant bacterial strains. Similar RI-based results as those in recent experiments were obtained when assessing Pheophorbide as an efflux pump inhibitor, as it promoted the more effective accumulation of the photosensitizer inside the bacterial cells [45].

Another promising strategy to combat resistant pathogens is the combination of PDT with traditional antibiotics, such as ciprofloxacin. PDT is a treatment modality that utilizes photosensitive compounds, which generate ROS when exposed to light of a specific wavelength in the presence of atmospheric oxygen. These highly reactive molecules damage bacterial cell membranes, proteins, and nucleic acids, ultimately leading to cell death [16,46]. PDT not only effectively inactivates bacteria but also limits the development of resistance, as bacterial mechanisms for adapting to ROS are highly constrained.

A key advantage of PDT is its localized action, enabling precise targeting of infected tissues while minimizing harm to healthy cells. PDT is effective against a broad spectrum of bacteria, including multidrug-resistant strains such as *S. aureus* and *E. coli*. Moreover, it is particularly effective in combating biofilms, which are a significant challenge for traditional antibiotic therapies [47].

The combination of ciprofloxacin with PDT can operate on multiple levels. First, PDT damages bacterial cell membranes, potentially facilitating the penetration of ciprofloxacin into the bacterial cell. This improved access allows the antibiotic to target DNA gyrase and topoisomerase IV more effectively. Additionally, PDT can disrupt bacterial defense mechanisms, such as efflux pumps, which are responsible for expelling antibiotics from the cell, thereby prolonging ciprofloxacin’s activity [47]. Importantly, combining ciprofloxacin with PDT may also reduce the risk of resistance development. Employing two distinct mechanisms to combat bacteria—chemical and photochemical—complicates the ability of pathogens to develop effective defenses against both simultaneously. Furthermore, the use of lower doses of ciprofloxacin in conjunction with PDT reduces selection pressure on bacteria, thereby limiting the emergence of resistant strains. The synergy between ciprofloxacin and PDT holds significant potential for enhancing the effectiveness of bacterial infection treatments. This is because their distinct mechanisms of action can mutually amplify their bactericidal effects.

In vitro studies have confirmed the effectiveness of the synergy between ciprofloxacin and PDT. Enhanced bacterial killing with ciprofloxacin/PDT combinations has been demonstrated for the Gram-positive *S. aureus* and *M. fortuitum* strains [48,49,50], as well as for Gram-negative strains, including the *Burkholderia cepacia* complex and *E. coli* [50,51].

Studies using planktonic cultures of *M. fortuitum*, *S. aureus*, *and E. coli*, as well as biofilm cultures of *S. aureus, E. coli*, and the *B. cepacia* complex, have demonstrated synergistic bactericidal effects when PDT is combined with ciprofloxacin [48,49,50,51].

We also observed a synergistic effect between ciprofloxacin and PDT. A significant antibacterial effect of PDT was demonstrated against *S. aureus* strains following 24 h of preincubation with ciprofloxacin. However, Gram-negative *E. coli* strains were not susceptible to the combined treatment with ciprofloxacin and PDT.

Similar to our findings, Ronqui et al. [50] investigated the synergistic antimicrobial effects of combining PDT with ciprofloxacin against *S. aureus* and *E. coli*, both in planktonic and biofilm forms. The results demonstrated that the combination significantly reduced planktonic bacterial counts and biofilm mass. The authors also examined whether the treatment sequence (PDT followed by ciprofloxacin or ciprofloxacin followed by PDT) influenced outcomes. They found that the synergistic effect was more pronounced when PDT preceded ciprofloxacin. PDT utilizes a photosensitizer activated by light to generate ROS, which disrupt bacterial cell membranes and biofilm structures. This disruption enhanced ciprofloxacin’s ability to penetrate bacterial cells and the biofilm matrix more effectively. The study showed that the combination of PDT and ciprofloxacin was significantly more effective than either treatment alone, particularly against biofilms, which are typically more resistant to standard antibiotic therapies. The findings suggest that combining PDT with ciprofloxacin could be a promising strategy for treating biofilm-associated infections, especially those caused by antibiotic-resistant bacteria. This synergistic approach may enhance the clinical efficacy of antibiotics such as ciprofloxacin, providing an effective solution for managing persistent infections where biofilm formation plays a central role in resistance.

Shih et al. [49] investigated the combined effects of PDT and ciprofloxacin in treating keratitis caused by *Mycobacterium fortuitum*. Keratitis resulting from *M. fortuitum* infections is difficult to treat due to biofilm formation and the related resistance of mycobacteria to antibiotics. The study demonstrated a synergistic effect between PDT and ciprofloxacin, suggesting that PDT could serve as a valuable adjuvant to antimicrobial therapy for intractable localized infections, such as infectious keratitis. Notably, sublethal concentrations of ciprofloxacin combined with PDT exhibited significantly greater bactericidal activity than ciprofloxacin alone.

Krespi et al. [48] evaluated the combined effects of ciprofloxacin and PDT on biofilms formed by methicillin-resistant *S. aureus* (MRSA). Biofilms, dense microbial communities encased within a protective extracellular matrix, significantly contribute to MRSA’s antibiotic resistance and persistence in chronic infections. The study revealed that ciprofloxacin alone had limited efficacy against MRSA biofilms, primarily due to poor penetration. However, the combination of ciprofloxacin with PDT yielded enhanced outcomes. PDT, which utilizes a photosensitizing agent activated by light exposure to produce ROS, effectively disrupted the biofilm matrix. This disruption facilitated deeper penetration of ciprofloxacin into the biofilm, allowing it to target bacteria that were previously protected. The synergistic interaction between PDT and ciprofloxacin resulted in a markedly improved bactericidal effect on MRSA biofilms compared to either treatment applied independently.

Cassidy et al. [51] investigated the effectiveness of combining PDT with ciprofloxacin to treat infections caused by the *Burkholderia cepacia* complex (Bcc), a group of antibiotic-resistant bacteria commonly associated with chronic lung infections in cystic fibrosis patients. The study found that while ciprofloxacin and PDT individually exhibited limited efficacy against Bcc biofilms, their combination produced a significant synergistic effect. Photodynamic antimicrobial chemotherapy (PACT), which employs a photosensitizer activated by light to generate ROS, disrupts the bacterial cell membrane and biofilm matrix, thereby increasing bacterial susceptibility to antibiotics. By compromising the structural integrity of the biofilm, PDT enhanced ciprofloxacin’s penetration through the biofilm layers, resulting in more effective bacterial eradication.

As described above, the antimicrobial efficacy of PDT/antibiotic combinations is strain-specific. Multiple studies have demonstrated that Gram-positive bacteria are generally more susceptible to combination treatments compared to Gram-negative bacteria. This difference is likely due to the structural differences in their cell walls, with the thick peptidoglycan layer in Gram-positive bacteria being more accessible to ROS generated during PDT, whereas the outer membrane of Gram-negative bacteria acts as an additional barrier, reducing susceptibility.

Research into the synergistic potential of plant metabolites in enhancing PDT highlights the ability of various herbal compounds to amplify their therapeutic effects. These metabolites can improve the cellular uptake and reactivity of photosensitizers, which are critical to the efficacy of PDT. For example, plant extracts containing photosensitizing agents can enhance the production of ROS, thereby increasing cell damage in targeted areas, such as tumor cells or bacterial infections. Among these metabolites, flavonoids have garnered particular attention for their capacity to boost ROS generation when activated by specific wavelengths of light. This property enhances the precision and efficacy of PDT, leading to improved treatment outcomes in both antimicrobial and anticancer applications [52,53].

The combination of mangostin with PDT shows promising results in the treatment of bacterial infections, especially those resistant to conventional antibiotics. Several mechanisms may account for the synergistic effect between mangostin and PDT. First, mangostin’s ability to disrupt bacterial cell membranes enhances bacterial permeability, facilitating the uptake of photosensitizers used in PDT. This membrane damage may allow deeper penetration and higher intracellular accumulation of the photosensitizer, amplifying the photodynamic effect and increasing ROS production. Second, mangostin’s antioxidant properties may help regulate the local inflammatory response induced by ROS [3]. While ROS generated during PDT effectively target bacteria, they can also cause collateral tissue damage and inflammation in the exposed area. Mangostin, acting as an antioxidant, may mitigate these inflammatory effects, making the combination of mangostin and PDT safer and more effective for therapeutic use.

As demonstrated in the current study, irradiation of samples containing *S. aureus* ATCC 29213, previously treated with α-mangostin, resulted in a growth reduction. However, this effect was not observed in the other bacterial strains tested.

Javaid et al. [52] explored the synergistic effects of combining PDT with plant extracts from *Thuja occidentalis*, *Moringa oleifera*, and *Solanum surattense*, and the chemotherapeutic agent doxorubicin hydrochloride (Dox-HCl), for the treatment of rhabdomyosarcoma. The study demonstrated a synergistic interaction between the medicinal plant-derived metabolites, Dox-HCl, and PDT, leading to enhanced therapeutic outcomes. Among the tested combinations, extracts from *M. oleifera* and *S. surattense* in conjunction with Dox-HCl and PDT exhibited greater synergistic effects compared to the *T. occidentalis* extract with Dox-HCl and PDT [52].

As demonstrated in the present study, irradiation of samples previously treated with the combined action of ciprofloxacin and mangostin reduced the survival of *S. aureus* strains but did not inhibit the growth of *E. coli* strains. This supports the findings of other researchers, who have concluded that Gram-negative strains are generally much more resistant to eradication, even when subjected to combined treatments.

## 4. Materials and Methods

### 4.1. Bacterial Strains

Two clinical uropathogenic *Escherichia coli* (*E. coli* 208) and *Staphylococcus aureus* (MRSA 101) strains were obtained from the collection of the Department of Biology and Medical Parasitology, Wroclaw Medical University, Poland. Based on the susceptibility profile, both strains were classified as multidrug-resistant (MDR). Moreover, two reference strains, i.e., *E. coli* ATCC 25922 and *S. aureus* ATCC 29213, were used as quality controls for antibiotic susceptibility testing. The strains were maintained on slopes containing a nutrient broth and glycerol in a final concentration of 40% and were stored at −20 °C.

### 4.2. Antimicrobial Agents

Ciprofloxacin lactate (Proxacin^®^; Polpharma, Warsaw, Poland) was used in the experiment. Alpha-mangostin (purity ≥ 98%) was purchased from Sigma-Aldrich (Merck Life Science, Poznań, Poland).

### 4.3. Photosensitizer and Light Source

The photosensitizer, Photolon (chlorin-e6 + polyvinylpyrrolidone; Belmedpreparaty, Minsk, Belarus), at a concentration of 100 μg/mL, was used in the study. The Polaris 2 biostimulation laser therapy device (ASTAR, Bielsko-Biała, Poland), with an LED applicator emitting red light (λ 650–660 nm; nominal power density of 0.08 W/cm^2^), was used as a light source.

### 4.4. Antimicrobial Susceptibility Testing

The antibiotic susceptibility of the tested strains was determined using the Kirby–Bauer disc diffusion method, according to the recommendations of the European Committee on Antimicrobial Susceptibility Testing (EUCAST) [21]. A bacterial suspension at a density of 0.5 in. McFarland (1–2 × 10^8^ CFU/mL) was applied with a sterile swab to plates of Mueller–Hinton agar (MHA, bioMérieux, Craponne, France). Antibiotic discs from Becton Dickinson were then applied to the plate and incubated for 18–20 h at 36 °C under aerobic conditions. Antibiotic sensitivity was determined by measuring the diameter of the bacterial growth inhibition zones. The results were interpreted using EUCAST breakpoint tables [21].

### 4.5. Bacterial DNA Isolation

The bacterial cultures were incubated overnight at 37 °C in 3 mL of LB broth. Genomic DNA extraction from these cultures was performed using the Genomic Mini AX Bacteria Spin Kit (A&A Biotechnology, Gdansk, Poland). The integrity and concentration of the extracted DNA were assessed by UV–Vis spectrophotometry using a NanoPhotometer NP80 (Implen, Munich, Germany) and fluorometric analysis using a Qubit 2.0 fluorometer with the Qubit™ 1X dsDNA High Sensitivity Assay Kit (Invitrogen, Life Technologies, Carlsbad, CA, USA).

### 4.6. Genome Sequencing

Whole-genome sequencing (WGS) of bacterial isolates was performed using nanopore sequencing technology (NST). Libraries were prepared using the Rapid Barcoding Kit 24 V14 (SQK-RBK114.24, Oxford Nanopore Technologies, Oxford, UK). Sequencing was performed on a MinION Mk1B instrument using a Flongle Flow Cell R10.4.1 (FLO-FLG114, Oxford Nanopore Technologies, UK) together with the Flongle Sequencing Expansion Kit (EXP-FSE002, Oxford Nanopore Technologies, Oxford, UK). Basecalling of the raw sequencing data was performed using Dorado 0.5.3 and the DNA model (dna_r10.4.1_e8.2_400 bps_sup@v4.3.0), with subsequent demultiplexing performed using the same tool. Read filtering was performed with a chopper [54], which yielded reads with a minimum Phred average quality score of 13 and a minimum length of 200 bp. Genome assembly was carried out using Flye 2.9.3-b1797 [55] and further polished using Medaka 1.11.3.

### 4.7. Identification of Antibiotic Resistance Genes

The presence of antibiotic-resistance genes was determined using ResFinder-4.5.0 (Center for Genomic Epidemiology, Kongens Lyngby, Denmark). The genomic sequences were uploaded to the ResFinder web service and the search was performed with default settings, using a minimum threshold of 90% identity and 60% coverage for resistance gene identification [56,57].

### 4.8. MIC Evaluation

The minimum inhibitory concentrations (MICs) of ciprofloxacin and α-mangostin were determined in Mueller-Hinton broth (MHB; Gdansk, Poland) according to the CLSI guidelines for broth microdilution susceptibility testing [58].

### 4.9. Preparation of Bacterial Suspension

The bacteria were suspended in 1 mL of MHB and incubated at 37 °C for 2 h in a Julabo SW-22 shaking water bath (DanLab, Bialystok, Poland). The bacterial suspensions were then centrifuged (4500 rpm/5 min) and suspended in PBS (phosphate-buffered saline) to reach a final density of 0.5 McFarland (1–2 × 10^8^ CFU/mL). The suspensions prepared in this way were used in all of the experiments.

### 4.10. Effects of Ciprofloxacin, α-Mangostin, and Their Combination on Bacterial Survival

The bacterial suspensions were cultured in Eppendorf tubes at 37 °C for 2, 4, 6, and 24 h in the MHB medium containing ciprofloxacin at a concentration of 0.5 × MIC, α-mangostin at 0.125 × MIC, or both agents at the concentrations mentioned above. The untreated (control) samples did not contain any antibacterial agent. The viable counts of bacteria were determined by the serial dilution method. The agar plates were incubated at 37 °C for 24 h and the bacterial colonies were counted and reported as colony-forming units per milliliter (CFU/mL).

### 4.11. Estimation of the Post-Treatment Effect of Ciprofloxacin, α-Mangostin, and Their Combination

To estimate the post-treatment effect (PTE), the strains were exposed to 0.5 × MIC of ciprofloxacin, 0.125 × MIC of α-mangostin, or 0.5 × MIC of ciprofloxacin with 0.125 × MIC of α-mangostin. The samples containing unexposed bacteria constituted the control. All the samples were shaken aerobically in a Julabo SW-22 water bath (DanLab, Bialystok, Poland) at 37 °C for 24 h. The antimicrobial agents were removed by washing with PBS and centrifugation (4000× *g* for 5 min). The control culture was also centrifuged. The treated and control cultures were placed in fresh MHB and incubated in a water bath at 37 °C for 4 h. Growth was monitored hourly by removing 100 μL samples, performing serial dilutions, and determining the number of CFU of the sample per mL on agar plates. PTE was defined as the time required to exceed the logarithmic value read at time t0, i.e., after the removal of antibacterial factors.

### 4.12. Quantitative Assessment of Photosensitizer Accumulation Inside Bacterial Cells

Digital holotomography (DHT) was used to evaluate the influence of α-mangostin on the penetration of bacterial cells using the Photolon photosensitizer (Belmedpreparaty, Minsk, Belarus). DHT, by allowing the reconstruction of the distribution of the 3D refractive index (RI), provides the quantitative measures that allow the study of changes in bacterial cell density. It has been shown that RI-based measures provide quantitative information on the effectiveness of photosensitizer accumulation inside bacterial cells [45,59], since the accumulation of the photosensitizers inside the cells leads to an increase in cell density. Therefore, the same protocol was used in a recent experiment to evaluate the possible influence of α-mangostin on the cell membrane permeability and the penetration of Photolon into the bacterial cells.

Two bacterial species from the ATCC collection (*E. coli* ATCC 25922 and *S. aureus* ATCC 29213) were used in this experiment. The ATCC controls were chosen because they are well-characterized in terms of their biological and genetic properties. This ensures that the results obtained are more standardized, making it easier to compare data from different studies. Clinical strains, on the other hand, may exhibit variability due to factors such as source of isolation or culture conditions, which could introduce unwanted variables and complicate the interpretation of results.

The examination was performed with the use of the DHT system (3D Cell Explorer, Nanolive, Tocholenz, Switzerland). For each bacterial sample (without Photolon and α-mangostin, with only Photolon, with only α-mangostin, and with both α-mangostin and Photolon), 15 to 20 3D-RI tomograms (each containing 96 2D-RI tomograms) were recorded. RI data processing was performed using Matlab software (Matlab 2022b, Mathworks, Natick, MA, USA). To numerically reconstruct the digital holograms, the reference RI measurement of the Mueller–Hinton liquid broth in which the bacterial samples were cultivated was performed using the Abbe refractometer (NAR-2T, minimum scale: 0.001, ATAGO Co., Ltd., Tokyo, Japan) at 20 °C. The numerical reconstruction of the 3D-RI tomograms and RI-histogram thresholding were performed using STEVE software (v. 1.6.3496, Nanolive, Ecublens, Switzerland). RI data processing, i.e., determination of the 2D maps of the maximal RI values and the segmentation of bacterial cells, as well as determination of the quantitative measures (average RI value, bacterial cell volume, and dry mass value), was performed using Matlab software (Matlab 2022b, Mathworks, Natick, MA, USA). The dry mass was evaluated based on the average RI value of bacterial cells in the following manner [60]:

$$drymass=\frac{V}{\alpha }\left(\frac{RI}{{RI}_{medium}}-1\right)$$

where α is the proportionality constant called the RI increment, RI is the average RI value of bacterial cells, RI medium is the average RI value of the medium (RI_medium_ = 1.337), and V is the cell’s volume. RI increment values vary in the range of 0.18 to 0.21 mL/g. In this model, the RI increment was assumed to be equal to 0.185 mL/g as the average value for proteins and nucleic acids. To confirm the presence of the statistically significant changes between the average RI values and dry mass values of bacterial cells treated only by Photolon and by both α-mangostin and Photolon, one-way ANOVA (significance level 0.05) was performed with the use of the Origin Software (OriginPRO 2023b, OriginLab Corporation, Northampton, MA, USA).

Additionally, a scanning confocal microscope (Leica TCS SPE, Leica, Wetzlar, Germany) was used to visualize photosensitizer penetration into bacterial cells and validate DHT results (Figure 4). Operating in fluorescence and DIC modes, it utilizes standard fluorescence lasers (405 nm, 488 nm, 532 nm, and 635 nm) and a halogen lamp for DIC. Imaging parameters (e.g., laser wavelength, pinhole diameter, and laser intensity) were software-controlled (LAS V.3.1). Bacterial samples (without Photolon and α-mangostin, with only Photolon, with only α-mangostin, and with both α-mangostin and Photolon) were imaged using an oil immersion objective (63×, NA = 1.3). Three-dimensional data were obtained by combining DIC and fluorescence images with X-Z and Y-Z cross-sections to localize the photosensitizer within cells. Fluorescence intensity in single-cell regions was measured to assess PS accumulation.

### 4.13. Experimental Conditions for the PDT

The 24 h bacterial suspensions containing ciprofloxacin at a concentration of 0.5 × MIC, α-mangostin at a concentration of 0.125 × MIC, ciprofloxacin with α-mangostin, and the control sample containing no antimicrobial agents (untreated) were plated in the wells of a 24-well plate. Then, the photosensitizer was added to the wells to obtain a final concentration of 100 µg/mL photosensitizer. The plate was pre-incubated with the Photolon in the dark for 15 min at room temperature before being irradiated. The light exposure was performed with a laser applicator emitting low-energy red light (Polaris 2, Astar, Bielsko-Biała, Poland; λ 650–660 nm; nominal power density 0.08 W/cm^2^) with a total energy dose of 14.0 J/cm^2^ (irradiation duration 175 s). The samples were then serially diluted and plated in triplicate on nutrient agar dishes (Biomed, Kraków, Poland) immediately after illumination, and at 60, 120, 180, and 240 min after irradiation [61]. The agar dishes were incubated at 37 °C for 24 h and the number of CFU/mL was counted.

### 4.14. Morphological Changes of Bacterial Cells

Bacterial suspensions treated with ciprofloxacin, α-mangostin, ciprofloxacin + α-mangostin, ciprofloxacin + PDT, α-mangostin + PDT, and ciprofloxacin + α-mangostin + PDT, as well as untreated controls, were air-dried on glass slides and stained with a 1% DAPI solution. Following 10 min of staining in the dark, the slides were rinsed with PBS to remove excess stain and observed using a Nikon Eclipse 400 microscope (Nikon, Tokyo, Japan) with JENOPTIK GRYPHAX V2.2.0 software. Morphological changes were analyzed under 1000× magnification.

### 4.15. Statistical Analysis

The nonparametric Kruskal–Wallis test followed by Dunn’s multiple comparison test was used. Statistical calculations were performed using Statistica 13.3. (Stat Soft, Kraków, Poland). All the experiments were performed in three independent replicates. All values are expressed as the means ± SDs. Values of *p* ≤ 0.05 were considered statistically significant.

## 5. Conclusions

In conclusion, the question posed in the title is intriguing and highlights a critical area of research in combating multidrug-resistant pathogens.

Alpha-mangostin and PDT can be adjuvants to ciprofloxacin for combating uropathogenic strains of *E. coli* and *S. aureus*. It has demonstrated significant synergy with ciprofloxacin in enhancing antibacterial activity against *S. aureus* strains. This effect may be attributed to α-mangostin’s disruption of bacterial membranes or interference with intracellular processes, which complements ciprofloxacin’s mode of action. However, α-mangostin does not appear to enhance ciprofloxacin’s activity against *E. coli* strains, likely due to differences in bacterial cell wall structure and resistance mechanisms.

This research highlights key aspects of using α-mangostin and PDT as potential adjuvants to ciprofloxacin for inactivating antibiotic-resistant Gram-positive and Gram-negative bacteria, which play a significant role in recurrent UTIs. Although the presented data are only preliminary and require further detailed investigations, they hold promise for scientists and clinicians aiming to develop innovative approaches to combat resistant infections. Collaborative efforts will be essential to translate these findings into tangible patient benefits. Future research should focus on evaluating the susceptibility of a broader range of bacterial strains to the tested antibacterial agents (ciprofloxacin, α-mangostin, and PDT), building on the current results to address existing knowledge gaps. Furthermore, additional research methods should be used to determine, in detail, the mechanism of action of α-mangostin on bacterial cells. Subsequent steps should include in vivo validation of in vitro findings and small-scale clinical trials, bringing this technology closer to practical applications for patients suffering from infections caused by antibiotic-resistant bacteria.

Despite the above-mentioned limitations, we believe our study provides a valuable foundation for future clinical research on the potential of PDT combined with conventional antimicrobials.

## Figures and Tables

**Table 1:** Molecular identification of resistance genes.

Resistance Gene	Predicted Resistance Phenotype	ResFinder Accession	Identity [%]
MRSA 101			
*blaZ*	Amoxicillin, Ampicillin, Penicillin, Piperacillin	CTTL01000039	100
*grlA* p. S80F	Ciprofloxacin	CP026964.1	99.5
*grlB* p. P585S			99.6
*gyrA* p. S84L			99.47
*mecA*	Amoxicillin, Ampicillin Amoxicillin + Clavulanic acid Ampicillin + Clavulanic acid Cefepime, Cefixime, Cefotaxime, Cefoxitin, Ceftazidime, Ertapenem, Imipenem, Meropenem, Piperacillin Piperacillin + tazobactam	NC_002951	99.95
*ermC*	Lincomycin, Clindamycin, Erythromycin, Quinupristin, Pristinamycin, Virginiamycin	M13761	100
***E. coli*** **208**			
*aph (6)-Id*	Streptomycin	CP000971	99.88
*aph (3″)-Ib*	Streptomycin	AF321551	100
*aadA5*	Spectinomycin, Streptomycin	AF137361	100
*blaCTX-M-27*	Amoxicillin, Ampicillin, Aztreonam, Cefepime, Cefotaxime, Ceftazidime, Ceftriaxone, Piperacillin, Ticarcillin	AY156923	100
*mph (A)*	Erythromycin, Azithromycin, Spiramycin, Telithromycin	D16251	100
*sul1*	Sulfamethoxazole	U12338	99.88
*sul2*	Sulfamethoxazole	AY034138	100
*tet (A)*	Doxycycline, Tetracycline	AJ517790	99.88
*dfrA17*	Trimethoprim	FJ460238	100
***S. aureus*** **ATCC 29213**			
*blaZ*	Amoxicillin, Ampicillin, Penicillin, Piperacillin	CZWI01000159	99.88

**Table 2:** Minimum inhibitory concentration (MIC) values (μg/mL) of ciprofloxacin and α-mangostin against the tested bacteria.

	Ciprofloxacin	α-Mangostin
MRSA 101	128 (r)	512
*E. coli* 208	64 (r)	512
*S. aureus* ATCC 29213	0.5 (s)	64
*E. coli* ATCC 25922	0.015 (s)	512

**Table 3:** PTE of ciprofloxacin, α-mangostin, and ciprofloxacin with α-mangostin.

	PTE Duration (Min)			
Treatment	*S. aureus* ATCC 29213	*E. coli* ATCC 25922	MRSA 101	*E. coli* 208
ciprofloxacin	195	60	0	60
α-mangostin	195	180	0	0
ciprofloxacin + α-mangostin	150	120	0	0

## Data Availability

The data presented in this study are available on request from the corresponding author.
